# Facile Determination of Sodium Ion and Osmolarity in Artificial Tears by Sequential DNAzymes

**DOI:** 10.3390/s17122840

**Published:** 2017-12-07

**Authors:** Eun Hye Kim, Eun-Song Lee, Dong Yun Lee, Young-Pil Kim

**Affiliations:** 1 Department of Life Science, Hanyang University, Seoul 04763, Korea; eun727712@hanmail.net (E.H.K.); eunsonamoo@naver.com (E.-S.L.); 2 Research Institute for Natural Sciences, Hanyang University, Seoul 04763, Korea; 3 Department of Bioengineering, Hanyang University, Seoul 04763, Korea; 4 Institute of Nano Science and Technology, Hanyang University, Seoul 04763, Korea; 5 Research Institute for Convergence of Basic Sciences, Hanyang University, Seoul 04763, Korea

**Keywords:** DNAzyme, osmolarity, sodium ion, salinity, tear, dry eye

## Abstract

Despite high relevance of tear osmolarity and eye abnormality, numerous methods for detecting tear osmolarity rely upon expensive osmometers. We report a reliable method for simply determining sodium ion-based osmolarity in artificial tears using sequential DNAzymes. When sodium ion-specific DNAzyme and peroxidase-like DNAzyme were used as a sensing and detecting probe, respectively, the concentration of Na^+^ in artificial tears could be measured by absorbance or fluorescence intensity, which was highly correlated with osmolarity over the diagnostic range (*R*^2^ > 0.98). Our approach is useful for studying eye diseases in relation to osmolarity.

## 1. Introduction

Tears are an attractive target for disease diagnosis because they exist as a clear fluid and are excreted in response to metabolic and environmental cues. Among eye diseases, dry eye is a commonly encountered symptom in humans and its incidence has increased considerably in recent years. Increased dryness in human tears can lead to eye diseases that damage the ocular surface and cause inflammation because of the instability of the tear film [1]. While individual objective tests prevail in the diagnosis of dry eye disease, tear osmolarity has been considered an alternative means for the rapid diagnosis of dry eye disease [2], as dry eye patients show increased tear osmolarity. In addition, it has been reported that ocular surface inflammation is accompanied by increased levels of osmolarity in the tear film of dry eye patients [3,4]. Osmolarity is a unit of osmotic concentration expressed as the number of osmoles (Osm) of solute per liter (L), and tear osmolarity is determined using an osmometers to measure the total concentration of all solutes including electrolytes in the tear fluid. Values below 290 mOsm L^−1^ are considered normal, while values above 316 mOsm L^−1^ indicate severe dry eye (hyperosmolarity) [5]. However, despite the potential application of osmolarity-based diagnosis, the current method for measuring osmolarity relies exclusively on the use of osmometer, which is a device operated by measuring either freezing point or osmotic pressure of a solution. Therefore, the development of a simple measurement of osmolarity is essential for those who have difficulty purchasing expensive equipment. To this end, we have noted that tear osmolarity is predominantly dependent on the concentration of sodium ion, which is the main electrolytes present in normal human tear fluid (120–170 mM), rather than potassium (6–42 mM), calcium (0.3–2 mM), and magnesium ions (0.3–1.1 mM) [6,7]. Although tear salinity contributes to nearly 90% of total tear osmolarity, few studies have examined the correlation between tear osmolarity and tear salinity.

Over the past decade, nucleic acid-based assays have been developed because of their high stability, easy preparation, and low cost [8,9,10]. Single-stranded DNA, such as aptamers and DNAzymes, can function in molecular recognition of a broad number of targets, enabling the development of practical assays without complicated instruments. Although many DNAzyme-based strategies are available for analyzing metal ions [11,12,13], no studies have detected salinity in tear samples using DNAzymes. Recently, a sodium ion-specific RNA-cleaving single-stranded DNAzyme (ssDNAzyme) was reported [14], which shows high selectivity for Na^+^ over a dynamic range (0.135–50 mM) and was used to image intracellular sodium ions in living cells by cleaving a fluorophore-quencher. Based on this study, we hypothesized that an improved design using this DNAzyme would be useful for rapidly determining tear salinity and osmolarity, as an alternative to the use of classical ion-specific electrodes or osmometers.

Here we report visual detection of sodium ion and relevant osmolarity in artificial tears using sequential DNAzymes. This method generates a colorimetric or a fluorescent signal via a sequential reaction of RNA-cleaving and peroxidase-like DNAzymes. In this regime, Na^+^-specific RNA-cleaving DNAzyme functions as a target recognition element, whereas peroxidase-like DNAzyme functions as a signal generator [15,16]. Sodium ion catalyzes the first DNAzyme, which releases a short DNA fragment (part of peroxidase-like DNAzyme). This results in full activation of peroxidase-like DNAzyme, leading to different detection signals by different substrates like classical peroxidase reactions. Because this output signal is proportional to the concentration of sodium ion as a major tear solute, we compared Na^+^-dependent signals with osmolarity measured in artificial tears.

## 2. Materials and Methods

### 2.1. Materials

Sodium chloride (NaCl, >99.5%), and potassium chloride (KCl, >99.5%) were purchased from Junsei Chemical (Tokyo, Japan). Hemin chloride (C_34_H_32_ClFeN_4_O_4_, >97%) was purchased from Sigma-Aldrich (St. Louis, MO, USA). 3,3′,5,5′-Tetramethylbenzidine (TMB) and QuantaRed™ substrate solution were purchased from Thermo Scientific (Waltham, MA, USA). Microplates (96-well clear or black) were purchased from Corning, Inc. (Corning, NY, USA). Oligonucleotides and modified oilgonucleotides were synthesized and purified by high-performance liquid chromatography or polyacrylamide gel electrophoresis (PAGE) by Integrated DNA Technology, Inc. (IDT, Coralville, IA, USA). Artificial tears (ophthalmic solutions, Hyundai Pharm. Co., Ltd., Seoul, Korea) were purchased from a local drug store in Seoul, Korea.

### 2.2. Preparation of DNAzyme-Based Assay

DNA sequences are presented in Table 1, where probe 1 is a sodium-specific DNAzyme, probe 2 including 3 guanines and internal ribonucleotide adenosine (rA) site is a complementary substrate for probe 1, probe 3 is a major part of peroxidase-like DNAzyme, and probe 4 is a complementary sequence for probe 3 and cleaved probe 2. The dsDNA complex (sodium ion-sensing part) was initially prepared in a similar manner as previously described [14]. Briefly, the complex was formed by annealing a mixture of probe 1 (5.45 µL at 10 µM) and probe 2 (4.55 µL at 10 µM) at a 1.2:1 molar ratio in reaction buffer (50 mM Bis-Tris containing 1 mM EDTA, pH 7.0) at room temperature (RT, 23–25 °C) for 1 h prior to use. As a sodium ion-specific DNAzyme control set, we used the reported NaA43E/S fluorogenic pair [14], where 5′-GCG GCG GTA CCA GGT CAA AGG TGG GTG AGG GGA CGC CAA GAG TCC CCG CGG TTA GAT AGA G-3′ (NaA43E) was labeled with Iowa Black FQ quencher at its 3′ end and 5′-CTC TAT CTA TrA GGA AGT ACC GCC GC-3′ (NaA43S) was 6-carboxyfluorescein fluorophore (FAM) and Iowa Black FQ quencher at its 5′ and 3′ end, respectively.

### 2.3. Gel Electrophoresis

The cleavage of the probe 1/probe 2 complex was verified by 6% native PAGE. Initially, the DNA complex (final 1 µM, 60 µL) was incubated for 1 h at RT with different metal ions (final 135 mM, 40 µL) or different concentrations of artificial tear solution (0–100%, 40 µL) in reaction buffer. The resulting product (9 µL) and 10× gel loading buffer (1 µL, TaKaRa) was loaded on a polyacrylamide gel. Gel electrophoresis was performed at 25 mA for 20 min in 1 × TB buffer (pH 8.0). After the gel was stained in a solution containing GelRed, the gel image was obtained using a UV-transilluminator (BioDoc-It^2^, UVP, Upland, CA, USA) at an excitation wavelength of 302 nm.

### 2.4. Detection of Salinity Using Dual DNAzymes

To determine salinity in buffer or artificial tears, 60 µL of the annealed complex (final 1 µM) was incubated with 40 µL of sodium ion solution or artificial tear solution in distilled water at RT for 1 h. A stock solution of sodium ion (135 mM) or artificial tear (containing 127 mM NaCl and 17 mM KCl) was diluted in the reaction buffer and prepared at final concentrations of 0–100% at 20% intervals. The resulting product (50 µL) was reacted with probe 3 (50 µL at 1 µM) and probe 4 (50 µL at 1 µM) by serial incubation of 5 min at 95 °C and 25 min at RT, which was followed by the addition of hemin solution (8 µL at 12.5 µM) and binding buffer (42 µL at 40 mM Tris containing 200 mM NaCl, 50 mM KCl, and 20 mM MgCl_2_) to the mixture at a final volume of 200 µL. This solution was incubated for 1 h at RT to generate a G-quadraplex structure. Next, to induce absorbance or a fluorescence signal, 100 µL aliquot of the final reactant was transferred into a 96-well microplate (clear plate for absorbance measurement or black plate for fluorescence measurement). Next, 100 µL of TMB-containing reaction solution (colorimetric substrate) or 100 µL of QuantaRed-containing reaction solution (fluorescence substrate) was added to each microplate well before recoding the signal change. Typically, the absorbance or fluorescence intensity was measured 15 min after the reaction using a multimode plate reader (Varioskan, Thermo Scientific, Inc., Waltham, MA, USA) and a time-dependent signal was acquired using the multimode plate reader every 5 min for 1 h at an excitation/emission of 560 nm/580 nm (for QuantaRed) or absorbance of 650 nm (for TMB). The color images of absorbance and fluorescence were acquired using a mobile camera and fluorescence imaging system in a black-chambered box (KIF-300, Korea Lab Tech, Korea), respectively.

### 2.5. Analysis of Osmolarity in Artificial Tear

The osmolarity of artificial tear was measured using a micro-osmometer (Fiske Model 210, Advanced Instruments, Norwood, MA, USA) via freezing-point depression. Typically, a 20 µL aliquot sample of artificial tear solution (0–100%) was measured in a disposable tube and the mean value was calculated from triplicate experiments.

## 3. Results and Discussion

### 3.1. Principle of Detection

**Figure 1:**
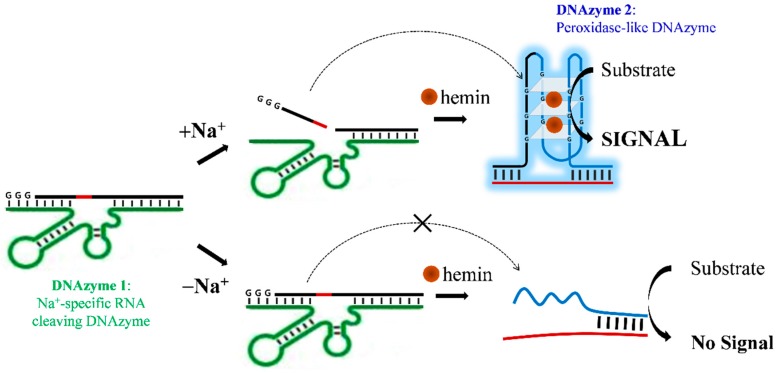
Schematic representation of DNAzyme-based detection of sodium ion (Na^+^). This assay consists of two different DNAzymes: DNAzyme 1 (Na^+^-specific RNA-cleaving DNAzyme) as a target recognition element and DNAzyme 2 (peroxidase-like DNAzyme) as a signal generator. The short DNA fragment released by Na^+^-triggered DNAzyme 1 fully activates DNAzyme 2 in the presence of hemin (co-factor) and the measurement mode can be varied depending on the substrates used.

As illustrated in Figure 1, sodium ions can be detected by a series of DNAzyme reactions. DNAzyme 1 (Na^+^-specific DNAzyme), a sequence modified by attaching CCC to the 3′-end of NaA43 *trans*-form reported previously [14], promotes the cleavage of an internal ribonucleotide adenosine (rA) site in the complementary strand upon the addition of Na^+^. This leads to the release of a short DNA fragment (a part of peroxidase-like DNAzyme including GGG), which can be assembled into whole DNAzyme 2 (peroxidase-like DNAzyme) in the complementary strand 2, where the apo-enzyme is activated in the presence of hemin (an iron-containing porphyrin derivative as a cofactor for DNAzyme), as reported previously [17,18,19]. It is important to note that the increased peroxidase activity of hemin by G-rich sequences has been found in telomeres, gene promoters and RNA transcripts, accounting for oxidative regulation of gene expression within cells [20]. By this principle, colorimetric or fluorogenic signals can be generated by DNAzyme 2 with different substrates. The sequences of used oligomer probes (1–4) are listed in Table 1. Because DNAzyme 2 can be reassembled from two pieces [21], we asymmetrically split the full sequence of peroxidase-like DNAzyme (5′-GGG TGG GTG GGT GGG TGG G-3′, 19 bp) into 5′-GGG-3′ (3 bp) and 5′-TGG GTG GGT GGG TGG G-3′ (16 bp), which were included in probe 2 and probe 3, respectively, to silence enzyme function under Na^+^-deficient conditions (bold gray color in Table 1). To induce a strong association between probe 3 and 4, a complementary sequence was appended to the 3’ end of probe 3. In this design, two DNAzyme reactions enable increase of Na^+^-specific signals and generation of different detection modes, which cannot be easily accomplished using a single DNAzyme.

### 3.2. Sensing Sodium Ions in Artificial Tear

**Figure 2:**
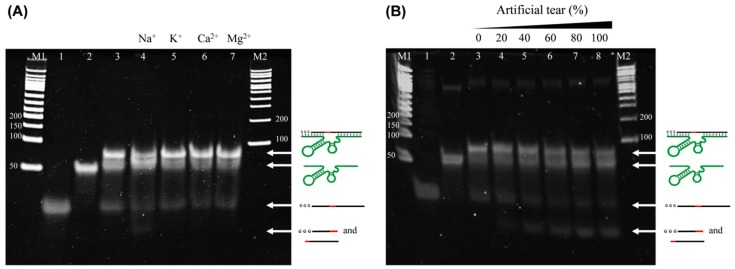
Gel electrophoretic images representing the cleavage of dsDNA (probes 1 and 2) at different ions (**A**) or at different concentrations of artificial tears (**B**). In (**A**) and (**B**), the first 3 lanes are the same; ssDNA probe 2 (lane 1), ssDNA probe 1 (lane 2), and dsDNA of probe 1/2 (lane 3). M1 and M2 indicate different DNA size markers. Lanes 4–7 in (**A**) represent the additions of Na^+^ (lane 4), K^+^ (lane 5), Ca^2+^ (lane 6), and Mg^2+^ (lane 7), whereas lanes 4–8 in (**B**) represent the additions of different concentrations of artificial tears (20, 40, 60, 80, and 100%). Gel electrophoresis was performed on a 6% native polyacrylamide gel at 25 mA for 20 min in 1 × TB and images were acquired on a UV transilluminator. The final concentration of monovalent and divalent ions was 135 mM.

We investigated the Na^+^-dependent cleavage of dsDNA complex by gel electrophoresis (Figure 2). In Figure 2A,B, when probe 1 (66 bp in lane 2) and probe 2 (30 bp in lane 1) were mixed, a strong new band corresponding to a dsDNA conjugate was observed, with a reduced intensity of each ssDNA (96 bp in lane 3). We incubated this assembly (probes 1 and 2) with different types of ions (Figure 2A) and different concentrations of artificial tears (Figure 2B). As a result, the cleaved band (~15 bp each from probe 2) was observed only in the presence of sodium ion, but not in the other samples (lanes 4–7 in Figure 2A). In addition, artificial tear also showed increased band intensity of the cleaved fragments as the band intensity of dsDNA decreased in a manner proportional to the concentration of artificial tears (lanes 4–8 in Figure 2B). Considering that the artificial tear used in this study contained 127 mM NaCl, 12 mM KCl, 2 mg mL^−1^ hypromellose (emulsifier), sodium hyaluronate (lubricant), phosphate ions, and sodium hydroxide, sodium ion was a major contributor to this reaction. Therefore, this result strongly indicates that sodium ion in the artificial tear enabled cleavage of the complex of probes 1/2, leading to a rapid release of the short DNA fragment from probe 2.

**Figure 3:**
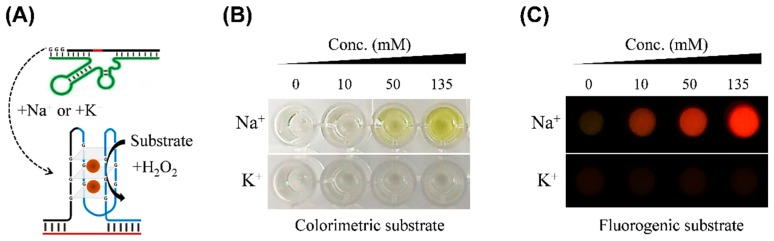
Na^+^-specific reaction of sequential DNAzymes with different detection mode: (**A**) schematic of DNAzyme reactions, (**B**) colorimetric analysis and (**C**) fluorescent analysis. Each signal by Na^+^ or K^+^ ions was compared as a function of concentration (0, 10, 50, and 135 mM).

Next, we examined whether DNAzyme 2 was activated by DNAzyme 1. As shown in Figure 3A, when the mixture of probe 1–4 was treated with K^+^ or Na^+^ at different concentrations (0, 10, 50, and 135 mM), the reaction was detected using TMB as a chromogenic substrate (Figure 3B) or QuantaRed as a fluorogenic substrate (Figure 3C) in the presence of H_2_O_2_. As the concentration of sodium ion increased, color intensity, which could be directly observed by eye, increased in yellow (top image in Figure 3B) and fluorescent emission also increased in red (top image in Figure 3C). In contrast, there were no significant signals in K^+^-treated samples (bottom images in Figure 3B,C). Notably, metal ions can affect the activity of peroxidase-like DNAzyme because the hemin-mediated G-quadruplex is formed in the presence of metal ions (particularly K^+^ ion) [11]. In addition, it was reported that Na^+^ ions can induce a conformational transition in the G-quadruplex, leading to reduced enzyme activity [12,22]. Our method avoids this problem as the tested ions were initially reacted with DNAzyme 1 in salt-free buffer, but not with DNAzyme 2. The detection limit of [Na^+^] was estimated to be 14.6 mM in absorbance and 0.4 mM in fluorescence based on 3× standard deviations of background signal, which is more improved to that of fluorogenic detection using Na^+^-specific DNAzyme [14]. Considering that the concentration of Na^+^ ions in human tears varies from 120 to 170 mM [6], the detection sensitivity of this assay is within an applicable range. This result demonstrates that full activation of split DNAzyme 2 was induced by sodium ion-triggered DNAzyme 1 reaction and that this method allows for different detection modes. The ability to exploit different measurement modes using same DNA probes can provide versatile strategy in measuring osmolarity. For example, colorimetric analysis can be used in places where equipment is not needed, and fluorescence analysis can be used effectively for more accurate and precise analysis.

**Figure 4:**
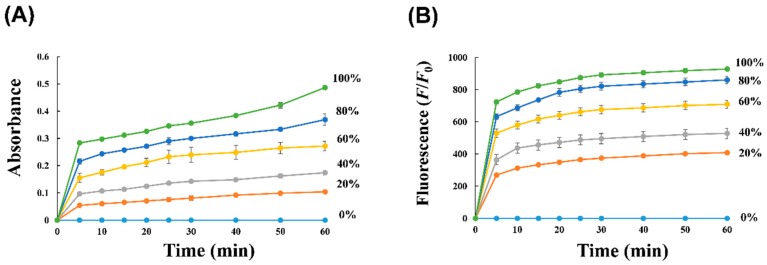
Time-dependent signal generation from sequential DNAzymes at different concentrations of artificial tears. The absorbance was obtained at 650 nm using colorimetric substrate (**A**) and the fluorescence signal was at excitation/emission wavelengths of 560 nm/580 nm using fluorogenic substrate (**B**), which monitored every 5 min for 1 h.

To gain an insight into the time-dependent reaction of artificial tears by DNAzymes, we tested various concentrations of artificial tears using sequential DNAzymes, wherein colorimetric or fluorescent signals were monitored using different peroxidase-reactive substrates over time (Figure 4). This experiment provides information on the minimum measurement time that current probes can detect [Na^+^] in tear sample and information on how fast the DNAzyme reacts with each substrate. When signal outputs were expressed as absorbance (using TMB in Figure 4A) or fluorescence (using QuantaRed in Figure 4B) with variable scales, the change in absorbance (A) or fluorescence (*F*/*F*_0_) was rapidly saturated with a steep initial slope. Half time at maximal values (*t*_1/2_) at different concentrations of artificial tears were determined to be 5–8 min as shown in Figure 4A and 2–3 min as shown in Figure 4B. This minor difference in *t*_1/2_ may be attributed to different substrate reactivities for peroxidase-like DNAzyme, but the overall range of *t*_1/2_ values was sufficiently rapid and similar to that by fluorogenic Na^+^-specific DNAzyme (data not shown), suggesting that the reaction of DNAzyme 1 was initially determined by the end product from DNAzyme 2. Importantly, the change in maximal equilibrium increased as the concentration of artificial tears increased, indicating that the cascade reaction by DNAzymes allows for rapid detection of sodium ions in artificial tears with versatile detection modes. Based on this result, we determined minimum reaction time (10–15 min) when the DNAzyme1/DNAzyme2 reactant produces colorimetric or fluorescent signal.

### 3.3. Correlation between Sodium Ion Concentration and Osmolarity in Artificial Tear

**Figure 5:**
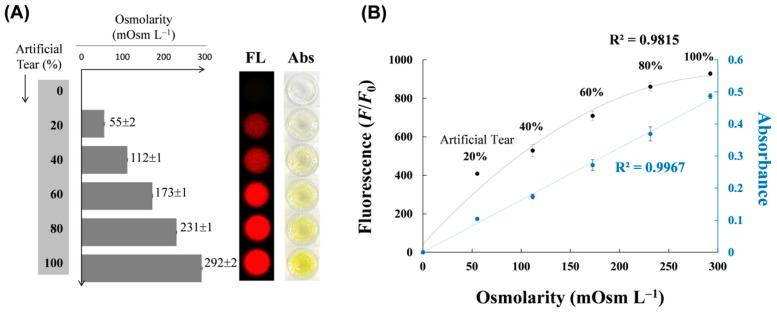
Relationship between osmolarity and sodium ions measured by DNAzymes in artificial tears. (**A**) Osmolarity (mOsm L^−1^) was displayed with gray bars as a function of artificial tear concentration, which was compared with fluorescent (red, left) and colorimetric image (yellow, right) via sequential DNAzyme reactions; (**B**) The plotted graph represents the relationship between osmolarity and each detection mode as a function of tear concentration. The standard deviation was obtained from three independent experiments.

Based on the above results, we examined whether sodium ion concentration was well-correlated with osmolarity in artificial tears (Figure 5). When artificial tears were diluted in distilled water and tested at 0–100% concentration, the osmolarity measured by the osmometer increased in proportion to the concentration, where 100% artificial tear solution (containing 127 mM sodium ions) showed a value of 292 mOsm L^−1^ (bar graph in Figure 5A). Similarly, the intensities of fluorescence and absorbance images measured by DNAzymes increased in the same concentration range (microplate images in Figure 5A). When two independent data sets were plotted as a function of tear concentration-based osmolarity, there was a hyperbolic dependence in fluorogenic detection (black line in Figure 5B) or linear dependence in colorimetric detection (green line in Figure 5B), with the high coefficients of determination (*R*^2^) accounting for the strongly positive relationship between osmolarity and both measurements. This result shows that DNAzyme-based detection of sodium ions in artificial tears is useful for estimating tear osmolarity. Theoretically, tear osmolarity did not represent only the concentration of sodium ions (1 mM NaCl solution generates 2 mOsm L^−1^) because many other ions or molecules (e.g., glucose, metabolite, and hormone) in tears contributed to total tear osmolarity. However, the change in tear osmolarity is likely proportional to that in sodium ions detected by DNAzymes because the amount of NaCl accounts for approximately 90% of total tear osmolarity. Notably, elevated osmolarity in tears is primarily related to increased levels of sodium chloride [23]. Particularly, considering that tear osmolarity in dry eye can reach more than 13–34 mOsm L^−1^ than that in normal tear drops [24,25], this change in osmolarity is equivalent to the signal change in the amount of 7–17 mM increase in sodium ions or 10% increase in artificial tears, supporting that our data can discriminate the dryness in tear drops by salinity or osmolarity.

Despite the availability of chemical [26,27,28,29] and electrical impedance-based assays [30] that generally detect sodium ions and osmolarity, DNAzyme-based Na^+^-assay has many advantages over classical methods. First, the rapid reaction rate (*t*_1/2_ < 10 min) and high specificity for sodium ions results in rapid and accurate measurement. In contrast, fluorescence-based chemical probes can induce slow reactivity (*t*_1/2_ > 30 min) and cross-reactivity among other metal ions, and other potentiometric ion sensors require sophisticated electrodes. Second, cost-effective and reproducible DNA-based designs allow the user to easily monitor tear osmolarity without the need for expensive osmometers or electric devices. Moreover, this method is not limited in fluorescence measurement. Various measurement modes can be used, including colorimetry, fluorescence, and chemiluminescence, which has great potential for versatile applications. Most significantly, unlike blood and urine, tear drops do not contain many biological components that may seriously affect sensing performance. Since tear samples can be directly added to our designed assays without pretreatment, this process is very attractive for in vitro diagnosis or non-invasive clinical tests. However, a small volume of real human tears at sample collection (a few microliters) is the main limitation of this method (typically, this method requires a relatively large volume of 40 µL) although the reaction volume can be reduced by using smaller biochip platforms and the sample can be diluted within a dynamic range. Additionally, in terms of total assay time, our method generally requires more than 1 h (serial reactions of DNAzyme 1, assembly, and DNAzyme 2 take at least 30, 30, and 15 min, respectively), which still needs to be reduced considering the relatively rapid measurement time (less than 10 min) of osmometer-based analysis [31]. By combining our strategy with microfluidics techniques that consume very small sample volumes with rapid assay time, this method will be practical for diagnosis using real samples. Taken together, current study suggest a new platform for measuring salinity-based osmolarity and is expected to be useful in future tear diagnosis.

## 4. Conclusions

We demonstrated the simple determination of sodium ion-based osmolarity in artificial tears using functional DNAzymes. By consecutively reacting two DNAzymes (Na^+^-specific DNAzyme and peroxidase-like DNAzyme), the concentration of sodium ions in artificial tears was quantitatively determined in colorimetric or fluorescent detection mode. When these detection methods were compared with osmolarity, there was a highly significant correlation between osmolality and [Na^+^] in artificial tears. Owing to high relevance of salinity, osmolarity and eye abnormality, our approach is useful for simple diagnostics of ocular diseases.

## Figures and Tables

**Table 1:** List of oligonucleotides used in the present study.

DNA	Characteristic	Sequence ^1^	Length (bp)
Probe 1	DNAzyme 1	5′–*GCG GCG GTA C*CA GGT CAA AGG TGG GTG AGG GGA CGC CAA GAG TCC CCG CGG T*TA GAT AGA GTT CCC*–3′	66
Probe 2	Complementary strand 1	5′–***GGG**AAC TCT ATC TA*T rAGG AA*G TAC CGC CGC*–3′	30
Probe 3	DNAzyme 2	5′–ATT ACA ATT ACT TAC TAA **TGG GTG GGT GGG TGG G**AA CTC TAT CTA T–3′	46
Probe 4	Complementary strand 2	5′–ATA GAT AGA GTT TTA GTA AGT AAT TGT AAT–3′	30

^1^ The complementary sequence between probe 1 and probe 2 is shown in italics and the complementary sequence between probes 2/3 and probe 4 is underlined. The peroxidase-like DNAzyme sequence is shown in bold.
